# Cone photoreceptor density in type I diabetic patients measured with an adaptive optics retinal camera

**Published:** 2019

**Authors:** Irina-Elena Cristescu, Florian Baltă, Leon Zăgrean

**Affiliations:** * Division of Physiology, Faculty of Medicine, “Carol Davila” University of Medicine and Pharmacy, Bucharest, Romania; ** Retina Clinic, Bucharest, Romania; *** Clinical Ophthalmology Emergency Hospital, Bucharest, Romania; **** Department of Ophthalmology, Faculty of Medicine, “Carol Davila” University of Medicine and Pharmacy, Bucharest, Romania

**Keywords:** adaptive optics, diabetes, photoreceptors

## Abstract

**Purpose:** To assess the variation in cone photoreceptor density on the basis of age compatibility between healthy subjects, on one side, and type 1 diabetic patients with no diabetic retinopathy, on the other.

**Methods:** A high resolution adaptive optics retinal camera in flood illumination regime was employed to image cones of 15 *type I diabetic patients* and 16 healthy controls. For each subject we scanned the cone mosaic in 4 perifoveal areas (nasally, temporally, superiorly and inferiorly) at 2, 3 and 4 degrees eccentricity. The impact of diabetes duration, gender and age were evaluated.

**Results:** In the type I diabetic group we found a meaningful lower cone density (p<0.05), except for *the temporal meridian at 2 and 4 * degrees eccentricity. Moreover, a significant asymmetry of cone photoreceptor densities was proved between the *horizontal and vertical meridians* in both diabetic and control groups.

**Conclusion:** The rtx1 retinal image evaluation demonstrated photoreceptors loss in DM1 diabetic patients prior to any clinical changes.

**Abbreviations:** AO = adaptive optics, SS = swept source, OCT = optical coherence tomography, BCVA= best corrected visual acuity, DM = diabetes mellitus, DR = diabetic retinopathy

## Introduction

*One of the* main causes of blindness worldwide is diabetic retinopathy (DR) is. Pathophysiological mechanisms of vision loss are now admitted to be generated by both microvascular complications and neuronal cells changes [**1**-**4**]. Therefore, the use of the term diabetic retinal disease instead of DR in order to incorporate both retinal vasculopathy and neuropathy was suggested [**5**]. Classically, it has been considered that DR is caused by the microvascular damage in the retina. Microaneurysms are regarded as the first visible clinical signs, whereas loss of pericytes, the first noticed histologic microvascular alteration [**5**-**7**]. Patients with DR may be asymptomatic *for a long time*, even until very late phases of the illness. Consequently, it is worldwide recommended that diabetic patients should be screened regularly and treated when needed. 

Additionally, the retina and the cerebral cortex have the same embryological origin; thus, in people with diabetes mellitus, functional retinal alterations may be linked to neurocognitive deficits[**8**]. There is evidence that retinal diabetic neuropathy (inner neuroretinal degenerations) may precede the diabetic retinal microvasculopathy [**9**]. Moreover, electrophysiology studies intimate that photoreceptors and retinal pigment epithelium show changes in diabetes. Also alterations of ionic transportation in photoreceptors and of oxidative stress were found in diabetic subjects [**10**]. Thus, studying photoreceptors changes might develop new biomarkers for the detection of retinal pathological changes in diabetic patients. 

As new state of art, imaging devices are available for clinicians, new approaches of early detection of pathological retinal changes can evolve. Adaptive optics (AO) ophthalmoscopy is now an accessible tool to visualize photoreceptors in the human living retina [**11**,**12**]. Several studies of human retinal photoreceptors in diabetic patients using the AO Imagine Eyes retinal camera have been achieved [**13**-**15**]. 

In our research, we have used an AO retinal camera to evaluate the cones parameters in adult patients with a history of type 1 diabetes and in adult healthy volunteers. Image acquisition was performed at 2, 3, and 4 degrees eccentricity from the fovea. AO imaging indicators (cone density, cone spacing and Voronoi diagrams) are able to reveal subtle changes of the parafoveal cones in DM1 patients before any clinical sign of retinopathy [**14**].

## Materials and methods

Designed in accordance to the Declaration of Helsinki, the present study was approved by the local ethics committee. All subjects offered their informed consent to participate in the study. All investigations are included in the screening protocol of diabetic retinopathy in the clinic. 

Study Participants. Patients with a medical history of type 1 diabetes mellitus and age-matched healthy subjects who received ophthalmological services at the Retina Eye Clinic were included in the study. The subjects were eligible if they satisfied the inclusion criteria: age over 18 years old, diagnosis of type I diabetes mellitus as defined by the American Diabetes Association from at least 1 year beforehand [**16**], with no diabetic retinopathy (according to the ETDRS scale [**17**]), 20/ 20 or better best corrected visual acuity (BCVA). Exclusion criteria were astigmatism higher than 2.50D, spherical errors higher than 3.00D, medical history of any ophthalmological pathology (including media opacity, macular edema, laser treatment, intravitreal injections, cataract surgery, or any other eye surgery). Control subjects were healthy, without any history of any ocular or systemic pathology. 

Examination. All subjects were performed the measurement of the best-corrected visual acuity on ETDRS charts, slit lamp eye exam of both the anterior and posterior segment and intraocular pressure measurement. Phenylephrine 10% and Tropicamide 1% were used to pharmacologically dilate the pupil in patients whose pupil diameter was less than 4.5mm. Comprehensive retinal imaging was achieved using the rtx1TM AO flood illumination retinal camera (Imagine Eyes, Orsay, France), SS OCT (DRI OCT Triton, Topcon), color fundus and red free photography (DRI OCT Triton, Topcon). Axial length determinations were achieved with optical biometry (Aladdin, Topcon). For further analysis, we have included results obtained from one eye of each subject. 

For the adaptive optics retinal images acquisition, the patients were asked to direct their eyes toward the internal yellow cross of the instrument whose coordinates were moved by the investigator. For one image with an improved signal to noise ratio, a set of 40 raw images acquired during 4 seconds were averaged in a 4 x 4 degrees field of view. Each examination scanned the cone mosaic in 4 perifoveal areas (nasally, temporally, superiorly and inferiorly) at 2, 3 and 4 degrees eccentricity, with a standardized 80 x 80 μm sampling window size. A comprehensive depiction of the rtx1 AO retinal camera and its applications are already available [**18**,**19**]. 

**Fig. 1:**
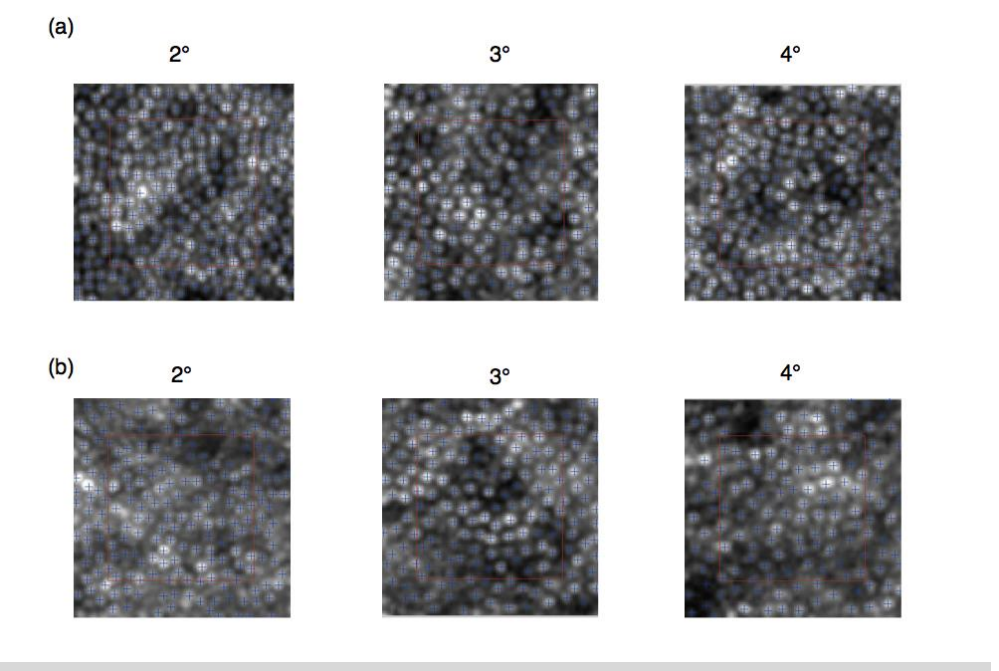
Images of regions of interest (red square) at 2, 3, and 4 degrees nasally analyzed in a control subject (a) and in a diabetic patient (b)

Image processing. The evaluation of the cone mosaic was accomplished with the software offered by the manufacturer, i2k Retina AO and AOdetect, respectively, Imagine Eyes, France (**Fig. 1**). The first one capacitates joining multiple images acquired using rtx1 AO retinal camera. Photoreceptors analyses done by AOdetect software provide the local mean cone density (cells/ mm2), inter-cone spacing (μm) and number of closest neighbours (Voronoi diagrams - %). 

Statistical analysis. Descriptive analyses of all variables with the outliers identification was achieved. For each subject four measurements (nasally, temporally, superiorly and inferiorly, respectively) have been obtained for each eccentricity (at 2, 3, and 4 degrees respectively). As assessed by Shapiro-Wilk’s test (p>.05), the parameters were normally distributed in both groups. Cones parameters in the two groups were compared using a T-test and one-way ANOVA. A simple linear regression was utilised to analyse the variation in cone density with duration of diabetes. Statistics were effectuated with the commercially available IBM SPSS Statistics software (version 23; Armonk, NY: IBM Corp). The maximum probability level accepted as statistically significant was 0.05. 

## Results

Participants. Thirty-one subjects were eligible for this study. Fifteen of them (5 females and 10 males) were patients with a diagnosis of type 1 diabetes mellitus and the other sixteen were healthy age-matched volunteers (10 males and 6 females). The BCVA of each subject was 20/ 20 or more. The mean ± standard deviation age in the diabetic group was 36.4 ± 6.46 and 39 ± 7.75 years old (p=0.32) in the control group, respectively (**Table 1**). 23.67 ± 0.82 was the mean ± standard deviation axial length in the diabetic group, whereas in the control group it was 23.93 ± 0.82 . The subjects in the DMI group have been affected by diabetes for 5 and 28 years (19.13 ± 7.47). 

**Table 1:** Characteristics of different age groups (mean ± standard deviation 95%CI)

	DM I group	Control group
N	15	16
Sex (female/ male)	5/ 10	6/ 10
OD/ OS	8/ 7	10/ 6
Age (years)	36.4 ± 6.46	39 ± 7.75
Group I (18-30 years)	4	3
Group II (31-40 years)	6	5
Group III (41-50 years)	5	5
Group IV (51-60 years)	0	1
Axial length (mm)	23.67 ± 0.82	23.93 ± 0.82
Duration of DM I (years)	19.13 ± 7.47	-

Cone metrics. **Table 2** contains the means, the standard deviations, the minimum and maximum values of cone densities measured at all eccentricities along all the meridians in both DM I and control study groups. There was no significant difference between the cone densities in the two studied groups at the temporal meridian, at 2 and 4 degrees eccentricity. The cone densities were significantly lower in the diabetic group at all locations, except the above-mentioned coordinates. The most important difference between the two groups has been acquired when we compared the average cone density values of each retinal eccentricity at the four quadrants.

**Table 2:** Mean ± standard deviation (minimum-maximum) cone density expressed in cone/ mm2 measured in the study and the control group at all eccentricities and at all meridians

Retinal Locations	2 degrees eccentricity (cones/ mm2)	3 degrees eccentricity (cones/ mm2)	4 degrees eccentricity (cones/ mm2)	average
Nasal				
Study group (DM1)	25346 ± 4233 (19049-33923)	22789 ± 2788 (19015-27614)	20211 ± 1641 (17966-23037)	22782 ± 3677
Control group	28521 ± 3634 (23607-36815)	26015 ± 2856 (21105-30905)	22638 ± 2378 (18204-26883)	25725 ± 3815
p	0.033	0.004	0.003	<0.001
Temporal				
Study group (DM1)	27182 ± 3440 (21559-31662)	25640 ± 2372 (21436-29913)	22492 ± 2140 (19494-25537)	25105 ± 3303
Control group	29160 ± 3042 (24582-33760)	27996 ± 3699 (22306-35407)	24092 ± 3227 (18892-30006)	27099 ± 3929
p	0.1	0.045	0.117	0.01
Superior				
Study group (DM1)	24159 ± 3600 (18219-30168)	24159 ± 3600 (18219-30168)	18315 ± 1262 (15385-20109)	21337 ± 3384
Control group	28535 ± 2906 (23268-34234)	23601 ± 2399 (19601-27217)	19716 ± 2025 (17317-23571)	23950 ± 4375
p	0.001	0.002	0.028	0.001
Inferior				
Study group (DM1)	24564 ± 3164 (20380-30401)	21979 ± 1611 (19885-24539)	18470 ± 1122 (15748-19704)	21671 ± 3284
Control group	21671 ± 3284	23575 ± 2473 (19179-26881)	19520 ± 1132 (17801-21470)	23725 ± 4161
p	0.003	0.043	0.015	0.01

In addition to this, a meaningful difference between the horizontal and vertical meridians at 3 and 4 degrees eccentricity has been discovered in the control group (**Table 4**). The same findings are available for the study group (DM1) in which we also found a significant difference at 2 degrees eccentricity between the horizontal and vertical meridians and at 3 and 4 degrees eccentricity between the nasal and temporal meridians (**Table 3**).

**Table 3:** T-test studying the differences between different meridians at all eccentricities in the study group (DM1)

Study group (DM1)	2 degrees eccentricity	3 degrees eccentricity	4 degrees eccentricity	average
N-T	0.203	0.005	0.003	0.002
S-I	0.746	0.102	0.724	0.45
H-V	0.047	<0.001	<0.001	<0.001

**Table 4:** T-test studying the differences between different meridians at all eccentricities in the control group

Control group	2 degrees eccentricity	3 degrees eccentricity	4 degrees eccentricity	average
N-T	0.593	0.1	0.157	0.089
S-I	0.656	0.97	0.738	0.797
H-V	0.491	<0.001	<0.001	<0.001

There were no significant correlations in both groups between cone photoreceptor density and age, gender or the duration of diabetes. 

## Discussions

In our research, we utilised rtx1 AO fundus camera to appreciate the differences of cone photoreceptor density in age-matched healthy subjects and type 1 diabetic patients. Cone density in the subject group (DM1, being diagnosed with diabetes 19.13 ± 7.47 years ago) was 10% lower than in controls. Nevertheless, there is a high variability of cone density among adult normal population and this difference cannot be regarded as clinically significant [**20**]. 

The first human retinal photoreceptors measurements are coming from post mortem histological analysis of human retinas [**21**]. It has been proved that in the centre of the fovea there is a density of 199000 cell/ mm2 that decreased to around 20000 cell/ mm2 within 1mm of the middle of the fovea.

Recently, advanced devices using the adaptive optics technology (AO scanning laser ophthalmoscopy and AO rtx1 retinal camera, Imagine Eyes) have disclosed relative databases of cone photoreceptors parameters of human subjects. Moreover, the connection between them and many variables (age, gender, axial length, refractive error, ocular dominance) was studied[**20**]. 

Our study confirmed previous findings of the cone parameters differences between diabetic patients and controls [**13**-**15**,**22**,**23**]. Lombardo et al. [**14**] have studied cone parameters differences at 1.5 degrees eccentricities in DM1 patients with no diabetic retinopathy or nonproliferative diabetic retinopathy and controls. Our research demonstrated similar differences of cone densities between DM1 patients and controls at 2, 3 and 4 degrees eccentricities and in all meridians (beside the temporal one at 2 and 4 degrees eccentricities). Nevertheless, Tan et al. [**24**] have found no differences of cone densities at 7 degrees eccentricity in DM1 patients and controls. As the author underlines, the short duration of diabetes (8.5 ± 4.1 years) in his DM1 group might have been an important factor.

Previous studies are confirming the cone photoreceptor densities at different eccentricities in the normal subjects' group [**20**,**25**,**26**]. 

The dissimilarity of the cone densities *between the nasal and temporal meridians at 3 and 4* degrees eccentricity in the study group is consistent with previous studies performed in normal subjects [**21**]. On the contrary, the normal subjects’ group didn’t present any asymmetry at any eccentricity. Nevertheless, we have found a significant higher density through the horizontal meridian than in the vertical one, in both groups (in the control group with an exception, at 2 degrees eccentricity, p=0.491). Thus, in the diabetic group, we found a difference of 8%, 11%, and 9% between horizontal and superior meridians at 2, 3, and respectively 4 degrees eccentricity. In the control group, we observed a difference of 12% and 16% at 3 and respectively 4 degrees eccentricity. These findings are congruent with other AO studies [**20**]. Moreover, we have found a higher asymmetry between the horizontal and vertical meridians at the same eccentricity in the control group in contrast to the study group (DM1). The way we use our vision might explain the higher density of cone photoreceptors through the horizontal meridian.When reading, our horizontal retina is used more than the vertical one. This hypothesis has been the subject of psychophysical studies which have proved that at a given eccentricity contrast sensitivity and spatial resolution are better through the horizontal than the vertical meridian. This fact is named the “horizontal-vertical anisotropy” [**27**]. Further studies including more subjects are needed to describe the cone parameters in diabetic and age-matched volunteers. 

Multifocal electroretinogram revealed functional deficits in diabetic patients [**28**]. The functional impairment might precede changes to photoreceptors integrity. Thus, the asymmetry between the horizontal and vertical meridians might become more consistent with increased diabetes duration. 

Nevertheless, this study has faced several limitations. Firstly, restrictions due to the constructive specificity of the camera, the resolution are not allowing the photoreceptors cone density assessment at the centre of the fovea. Consequently, the evaluation of a potential foveal cone loss in diabetic patients at this specific location is unlikely. Secondly, another weakness is the small number of selected retinal regions for cone parameters measurements within each imaged location. Thirdly, the small sample size is a weak point for this stage of the study. 

In conclusion, we have found significant differences in photoreceptor cone densities between controls and type 1 diabetic subjects with no clinical sign of diabetic retinopathy. Future studies with larger samples are required to supply databases that are more consistent. This might lead to more and more important information in the future for the early diagnosis of diabetic retinopathy, better comprehension of the consequences of the photoreceptors changes on the microvascular events and the visual function. 
